# Association of fluid balance trajectories with clinical outcomes in patients with septic shock: a prospective multicenter cohort study

**DOI:** 10.1186/s40779-021-00328-1

**Published:** 2021-07-06

**Authors:** Mei-Ping Wang, Li Jiang, Bo Zhu, Bin Du, Wen Li, Yan He, Xiu-Ming Xi, China Critical Care Sepsis Trial (CCCST) workgroup, Bin Du, Li Weng, Tong Li, Mei-Li Duan, Wen-Xiong Li, Bing Sun, Jian-Xin Zhou, Jian-Guo Jia, Xi Zhu, Qing-Yuan Zhan, Xiao-Chun Ma, Tie-He Qin, Shou-Hong Wang, Yu-Hang Ai, Yan Kang, Xue-Lian Liao, Xiang-Yuan Cao, Yu-Shan Wang, Du-Ming Zhu

**Affiliations:** 1 Department of Epidemiology and Health Statistics, School of Public Health, Capital Medical University, No.10, Xitoutiao, You’anmen, Beijing, Fengtai District China; 2 Department of Critical Care Medicine, Fuxing Hospital, Capital Medical University, No. 20, Street Fuxingmenwai, Beijing, Xicheng District China; 3 Department of Critical Care Medicine, Xuanwu Hospital, Capital Medical University, Beijing, 100053 China; 4 Medical Intensive Care Unit, Peking Union Medical College Hospital, Peking Union Medical College and Chinese Academy of Medical Sciences, Beijing, 100730 China

**Keywords:** Septic shock, Fluid overload, Group-based trajectory model, Clinical outcomes

## Abstract

**Background:**

Septic shock has a high incidence and mortality rate in Intensive Care Units (ICUs). Earlier intravenous fluid resuscitation can significantly improve outcomes in septic patients but easily leads to fluid overload (FO), which is associated with poor clinical outcomes. A single point value of fluid cannot provide enough fluid information. The aim of this study was to investigate the impact of fluid balance (FB) latent trajectories on clinical outcomes in septic patients.

**Methods:**

Patients were diagnosed with septic shock during the first 48 h, and sequential fluid data for the first 3 days of ICU admission were included. A group-based trajectory model (GBTM) which is designed to identify groups of individuals following similar developmental trajectories was used to identify latent subgroups of individuals following a similar progression of FB. The primary outcomes were hospital mortality, organ dysfunction, major adverse kidney events (MAKE) and severe respiratory adverse events (SRAE). We used multivariable Cox or logistic regression analysis to assess the association between FB trajectories and clinical outcomes.

**Results:**

Nine hundred eighty-six patients met the inclusion criteria and were assigned to GBTM analysis, and three latent FB trajectories were detected. 64 (6.5%), 841 (85.3%), and 81 (8.2%) patients were identified to have decreased, low, and high FB, respectively. Compared with low FB, high FB was associated with increased hospital mortality [hazard ratio (*HR*) 1.63, 95% confidence interval (CI) 1.22–2.17], organ dysfunction [odds ratio (*OR*) 2.18, 95% CI 1.22–3.42], MAKE (*OR* 1.80, 95% CI 1.04–2.63) and SRAE (*OR* 2.33, 95% CI 1.46–3.71), and decreasing FB was significantly associated with decreased MAKE (*OR* 0.46, 95% CI 0.29–0.79) after adjustment for potential covariates.

**Conclusion:**

Latent subgroups of septic patients followed a similar FB progression. These latent fluid trajectories were associated with clinical outcomes. The decreasing FB trajectory was associated with a decreased risk of hospital mortality and MAKE.

**Supplementary Information:**

The online version contains supplementary material available at 10.1186/s40779-021-00328-1.

## Background

Septic shock is a condition of hypotension and hypoperfusion that is induced by sepsis [1]. Although intensive care medicine is well advanced, severe sepsis and septic shock have a high prevalence and mortality rate [2, 3]. In high-income countries, approximately 6 million severe sepsis patients die annually [3]. In China, the mortality rate of severe sepsis and septic shock is greater than 50% in critically ill patients [4, 5]. International guidelines and studies support earlier intravenous fluid resuscitation for the management of sepsis or septic shock [1, 6]. However, critically ill patients seem to easily accumulate a positive fluid balance (FB) or fluid overload (FO) [7–10], and studies have demonstrated that a more aggressive course of fluid resuscitation is associated with adverse outcomes in critically ill subjects [8, 11].

Previous studies defined FO as a cumulative FB above a cutoff percentage of initial body weight over a certain period, 5% [12], 10% [11, 13] or any degree of positive fluid accumulation [14]. Furthermore, Woodward et al. [9] used a cubic spline model to assess the association between FO and major adverse kidney events (MAKE) in acute kidney injury (AKI) requiring continuous renal replacement therapy (RRT) and found a U-shaped nonlinear association, indicating that there may be a “sweet spot” for FB. The use of a single FB value for evaluating outcomes may not be adequate. Recently, a study on patients undergoing cardiac or aortic surgeries demonstrated that the trend of FB was associated with clinical outcomes [15]. Yende et al. [16] proclaimed that persistent elevation trajectories of inflammatory and immunosuppressive biomarkers were associated with higher long-term hospital readmission and mortality during hospitalization due to sepsis. However, studies on trends of FB in critically ill patients with septic shock have not been evaluated.

Our study used data from the China Critical Care Sepsis Trial (CCCST), which was a prospective multicenter cohort study. The aim of this study was to investigate the potential association between FB trends and clinical outcomes in critically ill patients with septic shock. We tested the hypotheses that there were latent trajectories of FB in those septic patients on day 7 and that the trajectories of FB were associated with hospital mortality and other clinical outcomes.

## Methods

### Study population

We derived data from the CCCST (China Critical Care Sepsis Trial), which was a prospective multicenter cohort study conducted at 18 ICUs of 16 tertiary hospitals from January 1, 2014 to August 31, 2015, and 4910 eligible adult patients were consecutively included. For patients with multiple admissions, only the first admission was included. Patients with septic shock who stayed in the ICU for three days or longer were included. We excluded patients with missing data on fluid input/output at initiation and those who had data for fewer than 2 fluid collection time points within 7 days during their ICU stay. Ultimately, 986 patients were enrolled in our analysis. This study is available from the Chinese clinical trials registry at www.chictr.org/cn/ (clinical trial number ChiCTR-ECH-13003934).

### Data collection

Standardized case report forms were used for data collection. A data surveillance panel was responsible for monitoring all patients who were sequentially included and checking the medical records for any missing or incorrect data. The patients’ demographics, source of admission, comorbid conditions, use of mechanical ventilation (MV) and RRT were documented. Clinical and laboratory values were used to calculate the severity of illness, Acute Physiology and Chronic Health Evaluation II (APACHE II) [17] and Sequential Organ Failure Assessment (SOFA) [18]. Serum creatinine (Scr), urine output, arterial partial oxygen pressure (PaO_2_), fraction of inspired oxygen (FiO_2_) and SOFA score were also continuously recorded for seven days or until discharge, whichever occurred earlier.

### Definition

FB was calculated using the following formula: FB = (total fluid input - total fluid output) ml/body weight initial (kg). Total fluid intake included all oral intake and intravenous fluid, which included resuscitation and maintenance fluids, blood products, drug infusions, and enteral and parenteral nutrition. Total fluid output included urine output, drainage fluid, ultrafiltration fluid and estimated gastrointestinal losses. Insensible loss was not considered in our study because of the difficulty of assessment. FO was defined as a cumulative FB (in liters) greater than 10% of the initial body weight [19]. Sepsis was defined as life-threatening organ failure caused by infection upon admission to the ICU or within the first 48 h after admission to the ICU, and septic shock was defined as sepsis associated with persistent hypotension that required vasoactive drugs to maintain a mean arterial pressure (MAP) ≥ 65 mmHg and had a serum lactate level > 2 mmol/L despite adequate volume resuscitation [20]. AKI and severity were categorized according to the Kidney Disease Improving Global Outcomes (KDIGO) guidelines [21]. We used an estimated baseline Scr or the lowest Scr value during the stay in the ICU, whichever was lower [22]. The simplified modification of diet in renal disease (MDRD) formula was used to estimate baseline Scr, which assumes a glomerular filtration rate (GFR) of 75 ml/min per 1.73 m^2^ and is customized for the Chinese population [23]. Acute respiratory distress syndrome (ARDS) was defined as PaO_2_/FIO_2_ < 300 mmHg according to the definition of Berlin [24]. Organ dysfunction-related adverse events were defined as an increase in the SOFA score compared with that at admission to the ICU. MAKE is a composite outcome, include new onset or sustained renal dysfunction, RRT-dependent discharge from the hospital, or an inability to recover to 1.5 times the baseline creatinine level. Severe respiratory adverse event (SRAE) was defined as ARDS or MV-dependent discharge from the hospital.

### Outcomes

Hospital mortality was the primary endpoint, which was defined as the status of patient survival before hospital discharge within 28 days after ICU admission. Organ dysfunction-related adverse events, MAKE and SRAE were secondary outcomes.

### Missing values

Overall, 5.6% of the clinical and laboratory data used to calculate the illness severity scores were missing in this study, and single imputation was performed for those variables with missing values. Weight data were missing for 2.1% of patients, and a mean weight of 65 kg was inputted; fluid data were missing in 4.0% of patients and were censored during the statistical analysis.

### Group-based trajectory model

A group-based trajectory model (GBTM) was used to identify latent subgroups of individuals following a similar progression of FB over the first 7 days of FB data. The GBTM is a specialized application of finite mixture modeling and is designed to identify groups of individuals following similar developmental trajectories [25]. This method assumes that the population is heterogeneous and is composed of a finite number of distinct groups. Model selection was assessed based on the Bayesian information criterion (BIC). BIC was used to determine the number of trajectories and the appropriate polynomial order of pattern (linear, quadratic or cubic) [25, 26]. The number of clusters was determined by a forward classifying approach, and lower BIC values were a better model fit. Furthermore, the sample size should include at least 5% of subjects, and the probability of each trajectory group was ≥0.70, defined as appropriated [27, 28]. GBTM was performed using the traj plugin in STATA to estimate the trajectory of FB [29].

### Statistical analysis

Continuous variables were represented as the mean ± standard deviation (SD) or the median with interquartile range (IQR), while categorical variables were represented as numbers with proportions. Differences among FB groups were compared by one-way analysis of variance or Wilcoxon rank-sum test for continuous variables, and the Chi square test for categorical variables. The Bonferroni post hoc test was used for multiple comparisons. Daily FB and cumulative FB between latent trajectory groups during the first 7 days following ICU admission are presented in box plots.

The association between latent trajectory groups and study outcomes (primary and secondary) was assessed by using univariate and multivariable forward stepwise Cox or logistic regression analysis with a cutoff of 0.1 for variables to enter the model and 0.05 to be removed. The likelihood ratio test was used to test the overall statistical significance of the Cox model. The variance inflation factors (VIFs) and tolerance coefficients were computed to test multicollinearity among the co-variates. Values of VIF exceeding 10 are often regarded as multicollinearity and were removed in the model. The covariates in the multivariable analysis were age, sex, APACHE II score and SOFA score on admission, source of admission, comorbidity condition (respiratory, cardiovascular, hypertension, chronic renal dysfunction, tumor and none), therapy (MV, RRT) and FO. In the multivariable analysis, the centers were included as a random effect. The hazard ratio (*HR*) or odds ratio (*OR*) and their 95% confidence interval (CI) were also reported. In the sensitivity analysis, we excluded patients with RRT during the first 7 days after ICU admission.

Analyses were performed using STATA version 15 (StataCorp, College Station, Texas, USA). A *P* value < 0.05 was considered to indicate statistical significance.

## Results

### Baseline characteristics between latent trajectory groups

**Fig. 1:**
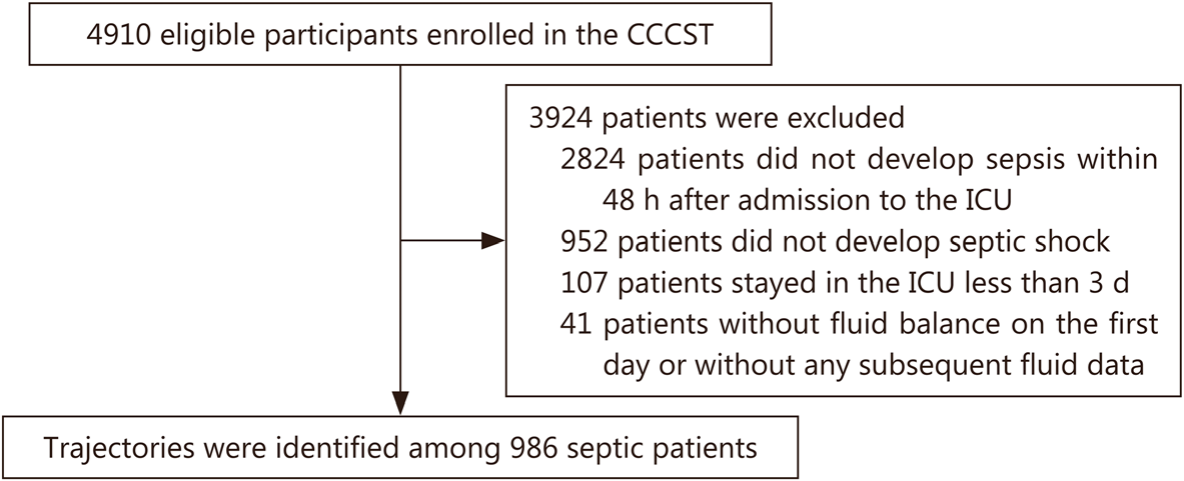
Flowchart of the patients included in the study. CCCST China Critical Care Sepsis Trial

A total of 4910 eligible participants were enrolled in the CCCST. After excluding 3924 patients who did not meet the inclusion criteria, 986 patients who developed septic shock during the first 48 h after ICU admission and stayed in the ICU for no less than 3 days were included (Fig. 1). Among those 986 patients, 624 (63.3%) were male with an average age of 63.7 (17.5) years. 659 (66.8%) came from medical wards, and 189 (19.2%) had no comorbidities. In addition, 83.8% of patients received MV, and 23.2% of patients received RRT (Table 1).

**Table 1:** Baseline characteristics of the study patients according to the 3 subgroups with different fluid balance trajectory patterns

Characteristics	All (*n* = 986)	Fluid balance trajectory patterns			*P*
		Decreasing FB (*n* = 64)	Low FB (*n* = 841)	High FB (*n* = 81)	
Fluid overload	198 (20.1)	33 (51.6)	87 (10.3) ^§^	78 (96.3)^§^	< 0.001
Age (year, $\overline{x}$ *± s*)	63.7 ± 17.5	66.3 ± 18.9	63.3 ± 17.3	65.3 ± 18.6	0.292
Sex [male, *n* (%)]	624 (63.3)	37 (57.8)	535 (63.6)	52 (64.2)	0.640
APACHE II ($\overline{x}$ *± s*)	22.1 ± 7.8	24.7 ± 7.4	21.6 ± 7.8^§^	25.4 ± 7.4	< 0.001
SOFA [M (IQR)]	8.0 (6.0–11.0)	11.0 (8.0–13.0)	8.0 (5.0–10.0)^§^	10.5 (8.0–13.0)^§^	< 0.001
Source of admission [*n* (%)]					0.006
Emergency department	175 (17.7)	12 (18.8)	143 (17.0)	20 (24.7)	
Medical	659 (66.8)	33 (51.6)	575 (68.4)^§^	51 (63.0)^§^	
Surgical	152 (15.4)	19 (29.7)	123 (14.6)^§^	10 (12.3)^§^	
Comorbidities [*n* (%)]					
Respiratory disease	76 (7.7)	5 (7.8)	64 (7.6)	7 (8.6)	0.946
Cardiovascular disease	155 (15.7)	11 (17.2)	130 (15.5)	14 (17.3)	0.862
Hypertension	326 (33.1)	19 (29.7)	284 (33.4)	23 (28.4)	0.518
Diabetes mellitus	192 (19.5)	9 (14.1)	167 (19.9)	16 (19.8)	0.528
Cancer	117 (11.9)	11 (17.2)	92 (10.9)	14 (17.3)	0.096
None	189 (19.2)	24 (37.5)	146 (17.7)^§^	19 (23.5)^§^	< 0.001
Treatments [*n* (%)]					
MV	826 (83.8)	62 (96.9)	691 (82.2)^§^	73 (90.1)^§^	0.002
Renal replacement therapy	229 (23.2)	12 (18.8)	178 (21.2)	39 (48.1)^§^	< 0.001

*FB* fluid balance, *SD* standard deviation, *IQR* interquartile range, *APACHE II* acute physiology and chronic health evaluation II, *SOFA* sequential organ failure assessment, *MV* mechanical ventilation, ^§^Compared with decreasing FB, *P* <  0.05

**Fig. 2:**
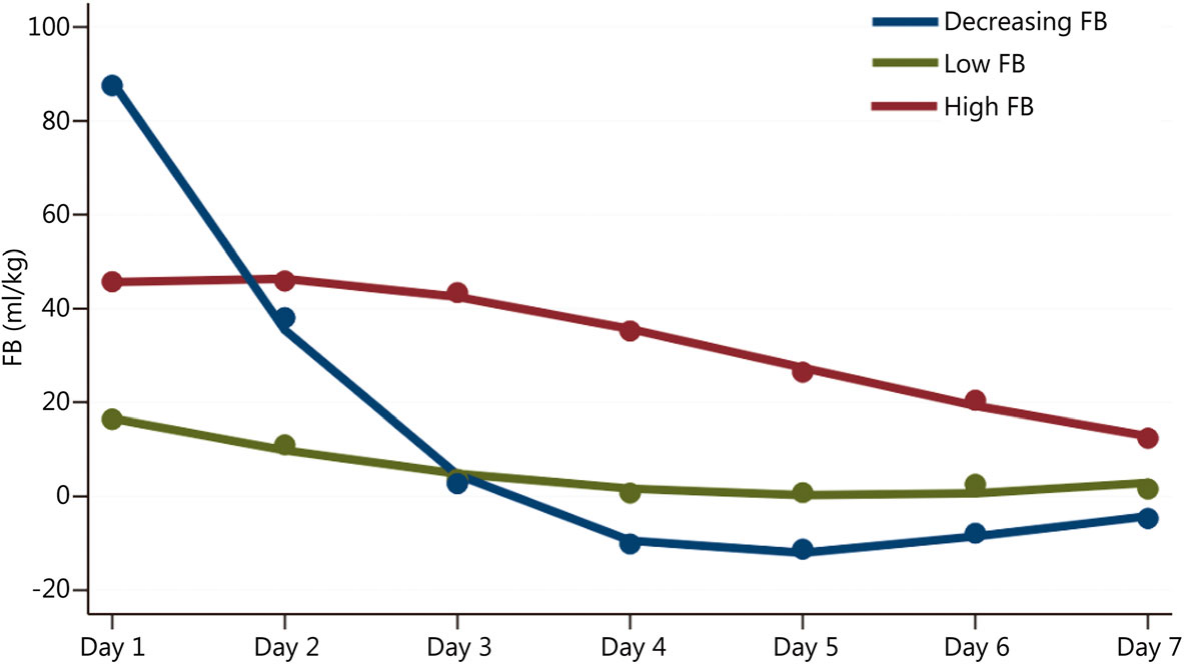
Fluid balance trajectory patterns during the first 7 days after admission to the ICU. FB fluid balance

Three FB trajectories reflected the potential pattern of decreasing, low or high fluid over 7 d (Fig. 2). Trajectory 1, described as “decreasing FB” (64, 6.5%), showed a trend in which the subjects started with a high FB and then decreased quickly from day 2 to day 3 and maintained a lower level. Trajectory 2, described as “low FB” (841, 85.3%), represented the subjects who maintained a low FB throughout the following 7 days. Trajectory 3, described as “high FB” (81, 8.2%), showed a trend in which subjects maintained a high FB from day 1 to day 3 and then slightly decreased from day 4. Although the age, sex and comorbidities in the three groups were not significantly different, the patients in the decreasing FB and high FB groups were likely to have higher severity of illness scores and receive more MV (Table 1).

### Daily and cumulative FB among the three trajectory groups

**Fig. 3:**
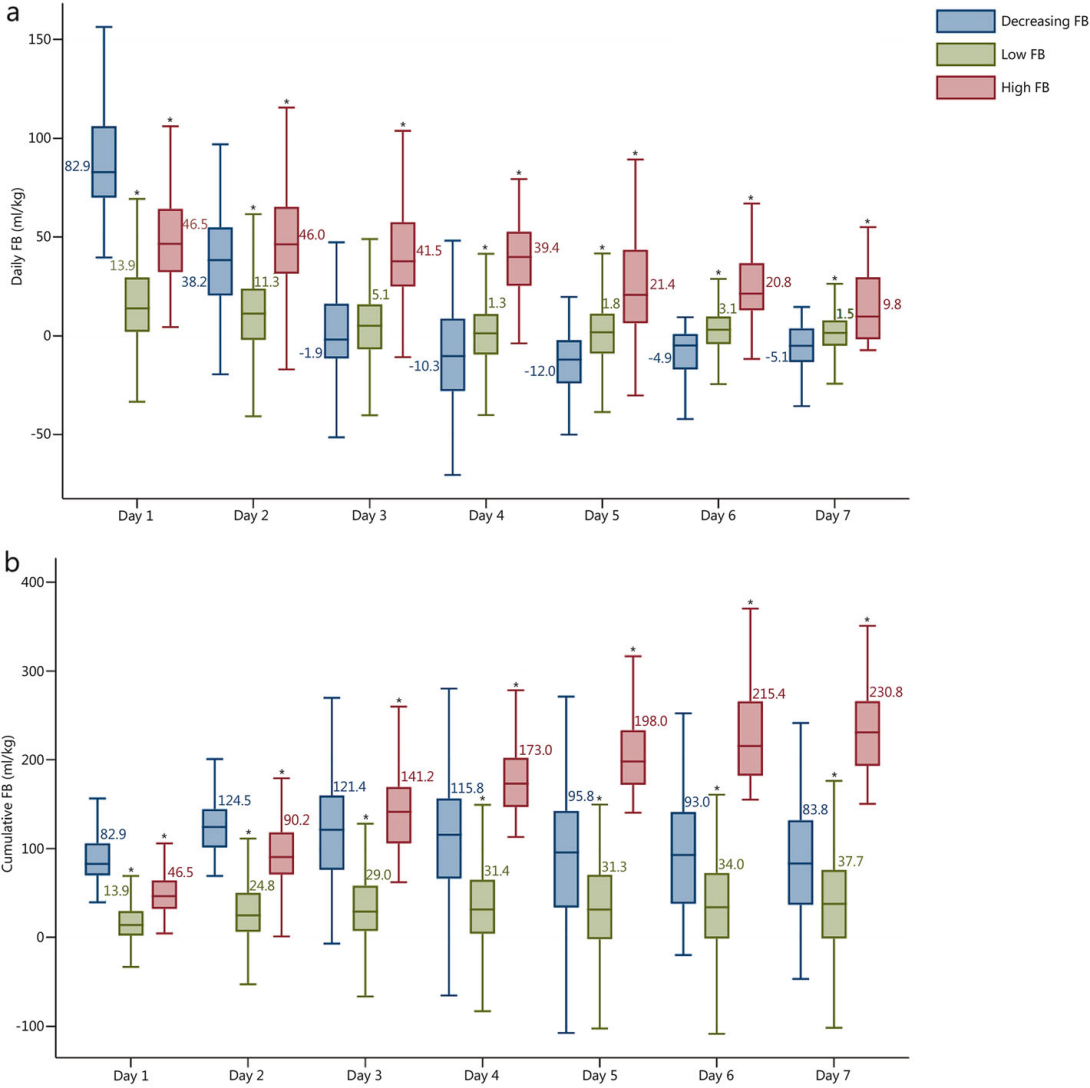
Daily and cumulative fluid balances in the three subgroups with different trajectory patterns. ^*^Compared with decreasing FB, *P* <  0.05; FB fluid balance

The three groups showed significantly different degrees of FB at all time points. The decreasing FB group had the highest daily FB on the day 1 of ICU admission, which then decreased rapidly to 38.2 ml/kg on the day 2 and remained in a negative FB from day 3 to day 7. The low FB group showed a slight change from 13.9 ml/kg on day 1 to 1.3 ml/kg on day 4 with a slight increase to 3.1 ml/kg on day 6. The high FB group also displayed a slightly decreased daily FB but maintained FB > 39.4 ml/kg during the first 4 days after ICU admission (Fig. 3a).

There was a gradual increase in fluid accumulation in the low FB group (from 13.9 ml/kg to 37.7 ml/kg) and a rapid increase in the high FB group (from 46.5 ml/kg to 230.8 ml/kg) following admission to the ICU. However, the decreasing FB group exhibited a progressive cumulative FB on the first 2 days (from 82.9 ml/kg to 124.5 ml/kg) after admission, which then decreased to 83.8 ml/kg on day 7 (Fig. 3b).

### Clinical outcomes

The clinical outcomes were shown in Table 2. 430 (43.6%) patients died in the hospital. Compared with low FB, the high FB had higher frequency of hospital mortality (71.6% vs. 46.9%), organ dysfunction (79.0% vs. 46.9%) and SRAE (58.0% vs. 29.7%). However, the decreasing FB had lower frequency of MAKE than the low FB (47.4% vs. 51.6%).

**Table 2:** Clinical outcomes of the study patients with different fluid balance trajectory patterns

Characteristics	All (*n* = 986)	Fluid balance trajectory patterns			*P*
		Decreasing FB (*n* = 64)	Low FB (*n* = 841)	High FB (*n* = 81)	
Clinical outcomes [*n* (%)]					
Hospital mortality	430 (43.6)	30 (46.9)	342 (40.7)	58 (71.6)^§^	< 0.001
Organ dysfunction	613 (62.2)	30 (46.9)	519 (61.7)	64 (79.0)^§^	< 0.001
MAKE	499 (50.6)	33 (51.6)	399 (47.4)^§^	67 (82.7)^§^	< 0.001
SRAE	373 (37.8)	19 (29.7)	307 (36.5)	47 (58.0)^§^	0.014
Length of stay [d, M (IQR)]					
Hospital^*^	20.0 (12.0–30.0)	21.0 (13.0–31.0)	19.0 (12.0–30.0)	20.0 (8.0–35.0)	0.576
ICU^*^	10.0 (5.0–16.0)	12.0 (8.0–17.0)	10.0 (5.0–16.0)	9.0 (4.0–23.0)	0.128

*FB* fluid balance, *IQR* interquartile range, *MAKE* major adverse kidney events, *SRAE* severe respiratory adverse events, ^*^For survivors; ^§^Compared with decreasing FB, *P* < 0.05

### Association of FO and FB trajectories with clinical outcomes

A total of 198 (20.1%) patients developed FO during the first 7 days after admission to the ICU. Compared with those without FO, patients with FO had a 1.4-fold increased risk of hospital mortality (*HR* 1.41, 95% CI 1.14–1.77, Supplemental file 1 Table S1). In addition, FO was an independent risk factor for kidney dysfunction and respiratory dysfunction (Supplemental file 1 Table S1).

Table 3 showed the association between the trajectory of FB and the clinical outcomes of septic patients. In adjusting for potential confounders, we found that compared with low FB, high FB was significantly associated with increased hospital mortality (*HR* 1.63, 95% CI 1.22–2.17), organ dysfunction (*OR* 2.18, 95% CI 1.22–3.42), MAKE (*OR* 1.80, 95% CI 1.04–2.63) and SRAE (*OR* 2.33, 95% CI 1.46–3.71), and decreasing FB was associated with lower risk of MAKE (*OR* 0.46, 95% CI 0.29–0.79). No significant association was observed between decreasing FB and outcomes of hospital mortality, organ dysfunction and SRAE.

**Table 3:** Hazard ratio or odds ratio for risks of clinical outcomes by the 3 subgroups of different fluid balance trajectory patterns

Clinical outcome	Events [*n* (%)]	Adjusted *HR*/*OR* (95% CI)	*P*
Primary outcome			
Hospital mortality			
Low FB	342 (40.7)	1.00	
High FB	58 (71.6)	1.63 (1.22–2.17)	0.001
Decreasing FB	30 (46.9)	0.86 (0.59–1.26)	0.331
Secondary outcome			
Organ dysfunction			
Low FB	519 (61.7)	1.00	
High FB	64 (79.0)	2.18 (1.22–3.42)	0.006
Decreasing FB	30 (46.9)	0.70 (0.40–1.02)	0.051
MAKE			
Low FB	399 (66.1)	1.00	
High FB	67 (82.7)	1.80 (1.04–2.63)	0.035
Decreasing FB	33 (51.6)	0.46 (0.29–0.79)	0.004
SRAE			
Low FB	307 (36.5)	1.00	
High FB	47 (58.0)	2.33 (1.46–3.71)	0.001
Decreasing FB	19 (29.7)	0.73 (0.42–1.28)	0.143

*FB* fluid balance, *MAKE* major adverse kidney events, *SRAE* severe respiratory adverse events, *CI* confidence interval, *OR* odds ratio, *HR* hazard ratio

### Sensitivity analysis

In the sensitivity analysis, we excluded 198 patients with RRT to eliminate the potential influence of RRT on the effect of FB. Nonetheless, we observed similar latent trajectories of FB (Supplement file 1 Fig. S1). There were 50 (6.3%), 685 (87.0%), and 53 (6.7%) patients identified with decreasing FB, low FB, and high FB, respectively. Using multivariable Cox or logistic regression analysis, we found that compared with the low FB group, the high FB group showed an association with an increased risk of hospital mortality, organ dysfunction, MAKE and SRAE, and the decreasing FB group showed an association with a decreased risk of organ dysfunction (Supplement file 1 Table S2).

## Discussion

In this prospective multicenter observational study, we used a GBTM to show the FB trends in septic patients and found that the trajectories of FB were associated with hospital mortality. Furthermore, we found that these trajectories were also associated with organ dysfunction, MAKE and SRAE during the first 7 days following ICU admission.

Fluid management is a cornerstone to maintaining primary hemodynamic stability, organs, and tissue perfusion and increasing oxygen delivery from the phases of salvage to de-escalation during septic shock [30, 31]. Several studies have shown that earlier resuscitation was associated with reduced mortality in sepsis or septic patients [6, 32–35]. However, excessive resuscitation can be harmful. Numerous studies have demonstrated that positive FB or FO was associated with increased adverse clinical events [14, 36–39]. Our previous study considered FO to be a fluid accumulation (L) greater than 10% of initial body weight (kg), which is associated with the occurrence of AKI and increases the severity of AKI [13]. FO is also a major predictor of poor clinical outcomes [12, 40–43]. A retrospective study showed that FO (≥ 10% L/kg) prolonged multiorgan failure based on the sub-SOFA score of the kidney in septic shock patients and increased 90-d mortality [44]. In our study, 20.1% of patients developed FO, and compared with patients without FO, those with FO had a 1.4-fold higher hospital mortality rate. In addition, FO status was an independent risk factor for MAKE and SRAE during the first 7 days after ICU admission in septic patients. However, in our study, there was no association between FO and organ dysfunction in the bivariate logistic regression.

There were also studies that used dose-response analysis to explore the effect of fluid on clinical outcomes [9, 14]. Garzotto et al. [14] concluded that the severity and speed of fluid accumulation were independent risk factors for mortality in critically ill patients and that FO was not only a value above a cutoff point but also any degree of positive FB. Woodward et al. [9] used cubic spline to assess the relation between FO and MAKE in critically ill patients with AKI requiring RRT and found a U-shaped nonlinear relation between FO and MAKE. This nonlinear finding suggests that there may be an optimal level of FB, and both below and above this optimal level may be harmful. Balakumar et al. [45] and Myles et al. [46] have confirmed this view.

However, the severity or the speed or the nonlinear association of fluid accumulation may not provide enough information on the effect of fluid on outcomes. Recently, a prospective study also showed that latent trajectories of FB (adjusted by body weight) in the first 72 postoperative hours of cardiac and aortic surgery were significantly associated with risk of AKI and dialysis. Patients with progressively positive FB had a 7.1-fold increased risk of AKI [15]. In our study, we used GBTM to explore the association between FB in the first 7 days after ICU admission and clinical outcomes in patients with septic shock and found that there were three latent trajectories of FB: Decreasing FB, low FB and high FB. The FB of the decreasing FB group on the first day of ICU admission was as high as 83 ml/kg and then rapidly decreased to 38 ml/kg on the second day. The peak volume of cumulative FB was 124 ml/kg on the second day. The low FB group maintained a lower FB status. The patients in the high FB group had a higher FB, above 40 ml/kg during the first 4 days following admission, and a cumulative FB as high as 230 ml/kg on day 7. Compared with the low FB group, the high FB group had an increased risk of hospital mortality, organ dysfunction, MAKE and SRAE. Decreasing FB was associated with a decreased risk of MAKE, although nearly half of the patients in the decreasing FB group developed FO. The difference among the potential trajectories of FB may be a better index and preferably reflect the whole condition in septic patients. In addition, there was a trend of a decreased risk of hospital mortality and organ dysfunction in decreasing FB patients, but these results were not statistically significant. Even though we excluded the effect of RRT on FB, three similar latent trajectories of FB were also detected. This interesting finding appears to be logical because earlier, sufficient fluid resuscitation can improve hypovolemia and tissue or organ hypoperfusion, which are associated with lower risk of mortality and other clinical outcomes.

In the multivariable analysis, we also found that a high FB trajectory was significantly associated with an increased risk of hospital mortality. These findings are similar to previous studies; earlier resuscitation was associated with a decreased risk of in-hospital mortality in severe sepsis and septic patients [6, 32]. FB in the first 24 h was not associated with an increased risk of mortality [11].

Our study was the first to investigate the effect of FB trajectories on clinical outcomes in septic shock patients. There are still several limitations in our study. First, the observational study design could not explain the causal relationships between trajectories of FB and outcomes. Second, we may have failed to adjust for other potential confounders in this observational study. Third, we did not consider the fluid input and output before ICU admission or in the operating theater, which cannot be ignored. Fourth, we failed to record diuretic use, which may influence fluid management and outcomes [47]. Furthermore, we used the lowest creatinine level during the ICU stay or the MDRD formula to estimate baseline creatinine, which may not reflect the true baseline creatinine value. The results of this study need to be further validated in studies with larger sample sizes.

## Conclusions

In critically ill patients with septic shock, latent trajectories of FB were associated with clinical outcomes. Decreasing FB in patients was associated with a decreased risk of hospital mortality and major MAKE. This finding suggested that in addition to the severity of fluid accumulation, a variety of daily FB trends were also associated with clinical outcomes in septic patients. However, this effect of the dynamic trend of FB on clinical outcomes needs further studies to be confirmed.

## Supplementary Information

**Additional file 1 Supplemental file 1: Table S1.** Fluid overload and clinical outcomes. **Table S2.** Hazard ratio (*HR*) or odds ratio (*OR*) for risks of clinical outcomes by the 3 subgroups of different fluid balance trajectory patterns in septic patients without RRT.

**Additional file 2: Fig. S1.** Fluid balance trajectory patterns in septic patients without RRT during the first 7 days after ICU admission.

**Additional file 3: Supplemental file 2.** All other ethical bodies that approved our study in the various centers involved.

## Data Availability

The datasets generated and or analyzed during the current study are available from the corresponding author upon reasonable request.

## Abbreviations

CCCST: China Critical Care Sepsis Trial
ICU: intensive care unit
FO: fluid overload
FB: fluid balance
GBTM: group-based trajectory model
MAKE: major adverse kidney events
SRAE: severe respiratory adverse events
*HR*: hazard ratio
*OR*: odds ratio
CFB: cumulative fluid balance
AKI: acute kidney injury
CRRT: continuous renal replace therapy
MV: mechanical ventilation
APACHE II: Acute Physiology and Chronic Health Evaluation II
SOFA: Sequential Organ Failure Assessment
SCr: serum creatinine
PaO_2_: arterial partial oxygen pressure
FiO_2_: fraction of inspired oxygen
MAP: mean arterial pressure
MDRD: simplified modification of diet in renal disease
GFR: glomerular filtration rate
ARDS: acute respiratory distress syndrome
BIC: bayesian information criterion
SD: standard deviations
IQR: interquartile ranges
CI: confidence interval
