# Supplementary material for: Association of fluid balance trajectories with clinical outcomes in patients with septic shock: a prospective multicenter cohort study
Source: Mil Med Res. 2021 Jul 6;8:40. doi: 10.1186/s40779-021-00328-1 (PMC8258941; doi:10.1186/s40779-021-00328-1)
Supplement: Supplementary file 1 — Additional file 1 Supplemental file 1: Table S1. Fluid overload and clinical outcomes. Table S2. Hazard ratio (HR) or odds ratio (OR) for risks of clinical outcomes by the 3 subgroups of different fluid balance trajectory patterns in septic patients without RRT. [file 40779_2021_328_MOESM1_ESM.docx]

**Table S1 Fluid overload and clinical outcomes**

| Clinical outcomes | Subgroups | Events [*n* (%)] | Un-adjusted *HR*/*OR* (95% CI)^#^ | *P* value | Adjusted *HR*/*OR* (95% CI)^#^ | *P* value |
| --- | --- | --- | --- | --- | --- | --- |
| **Primary outcome** |  |  |  |  |  |  |
| Hospital mortality | FO < 10% | 305 (38.7) | 1.00 |  | 1.00 |  |
|  | FO ≥ 10% | 125 (63.1) | 1.63 (1.32 - 2.01) | < 0.001 | 1.41 (1.14 - 1.77) | 0.002 |
| **Secondary outcome** |  |  |  |  |  |  |
| Organ dysfunction | FO < 10% | 481 (61.0) | 1.00 |  | - |  |
|  | FO ≥ 10% | 132 (66.7) | 1.28 (0.92 - 1.77) | 0.145 | - |  |
| MAKE | FO < 10% | 503 (63.8) | 1.00 |  | 1.00 |  |
|  | FO ≥ 10% | 153 (77.3) | 1.93 (1.24 - 2.77) | < 0.001 | 1.61 (1.09 - 2.38) | 0.016 |
| SRAE | FO < 10% | 284 (36.0) | 1.00 |  | 1.00 |  |
|  | FO ≥ 10% | 89 (44.9) | 1.62 (1.11 - 2.36) | 0.012 | 1.45 (1.02 - 2.05) | 0.021 |
| *FO* fluid overload, *CI* confidence interval, *MAKE* major adverse kidney events, *SRAE* severe respiratory adverse events, *HR* hazard ratio, *OR* odds ratio. ^#^*HR* for primary outcome, while *OR* for secondary outcomes. | | | | | | |

**
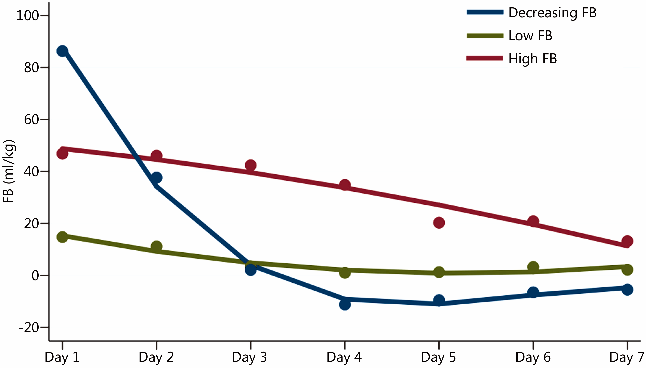
**

**Fig. S1** Fluid balance trajectory patterns in septic patients without RRT during the first 7 days after ICU admission

**Table S2. Hazard ratio (*HR*) or odds ratio (*OR*) for risks of clinical outcomes by the 3 subgroups of different fluid balance trajectory patterns in septic patients without RRT**

| Clinical outcomes | Subgroups | Events [*n*(%)] | Un-adjusted *HR*/*OR* (95% CI)^#^ | *P* value | Adjusted *HR*/*OR* (95% CI)^#^ | *P* value |
| --- | --- | --- | --- | --- | --- | --- |
| **Primary outcome** |  |  |  |  |  |  |
| Hospital mortality | Low FB | 242 (35.3) | 1.00 |  | 1.00 |  |
|  | High FB | 34 (64.2) | 2.14 (1.49 - 3.07) | < 0.001 | 1.58 (1.08 - 2.31) | 0.018 |
|  | Decreasing FB | 22 (44.0) | 1.16 (0.75 - 1.79) | 0.518 | 0.76 (0.49 - 1.20) | 0.244 |
| **Secondary outcome** |  |  |  |  |  |  |
| Organ dysfunction | Low FB | 408 (59.6) | 1.00 |  | 1.00 |  |
|  | High FB | 34 (64.2) | 2.24 (1.67 - 3.17) | 0.032 | 2.03 (1.58 - 2.89) | 0.009 |
|  | Decreasing FB | 17 (34.0) | 0.41 (0.22 - 0.78) | 0.007 | 0.35 (0.19 - 0.64) | 0.001 |
| MAKE | Low FB | 403 (58.8) | 1.00 |  | 1.00 |  |
|  | High FB | 42 (79.2) | 2.08 (1.09 - 3.97) | 0.026 | 1.95 (1.04 - 3.66) | 0.038 |
|  | Decreasing FB | 21 (42.0) | 0.54 (0.36 - 0.89) | 0.003 | 0.65 (0.36 - 1.15) | 0.136 |
| SRAE | Low FB | 232 (33.9) | 1.00 |  | 1.00 |  |
|  | High FB | 29 (54.7) | 2.36 (1.34 - 4.15) | 0.003 | 2.69 (1.45 - 5.01) | 0.002 |
|  | Decreasing FB | 13 (26.0) | 0.69 (0.36 - 1.32) | 0.257 | 0.74 (0.37 - 1.45) | 0.387 |
| *RRT* renal replacement therapy, *FB* fluid balance, *MAKE* major adverse kidney events, *SRAE* severe respiratory adverse events, *CI* confidence interval, *HR* hazard ratio, *OR* odds ratio. ^#^*HR* for primary outcome and death, while *OR* for secondary outcomes. | | | | | | |
