# Supplementary figures and images for: Association of fluid balance trajectories with clinical outcomes in patients with septic shock: a prospective multicenter cohort study
Source: Mil Med Res. 2021 Jul 6;8:40. doi: 10.1186/s40779-021-00328-1 (PMC8258941; doi:10.1186/s40779-021-00328-1)

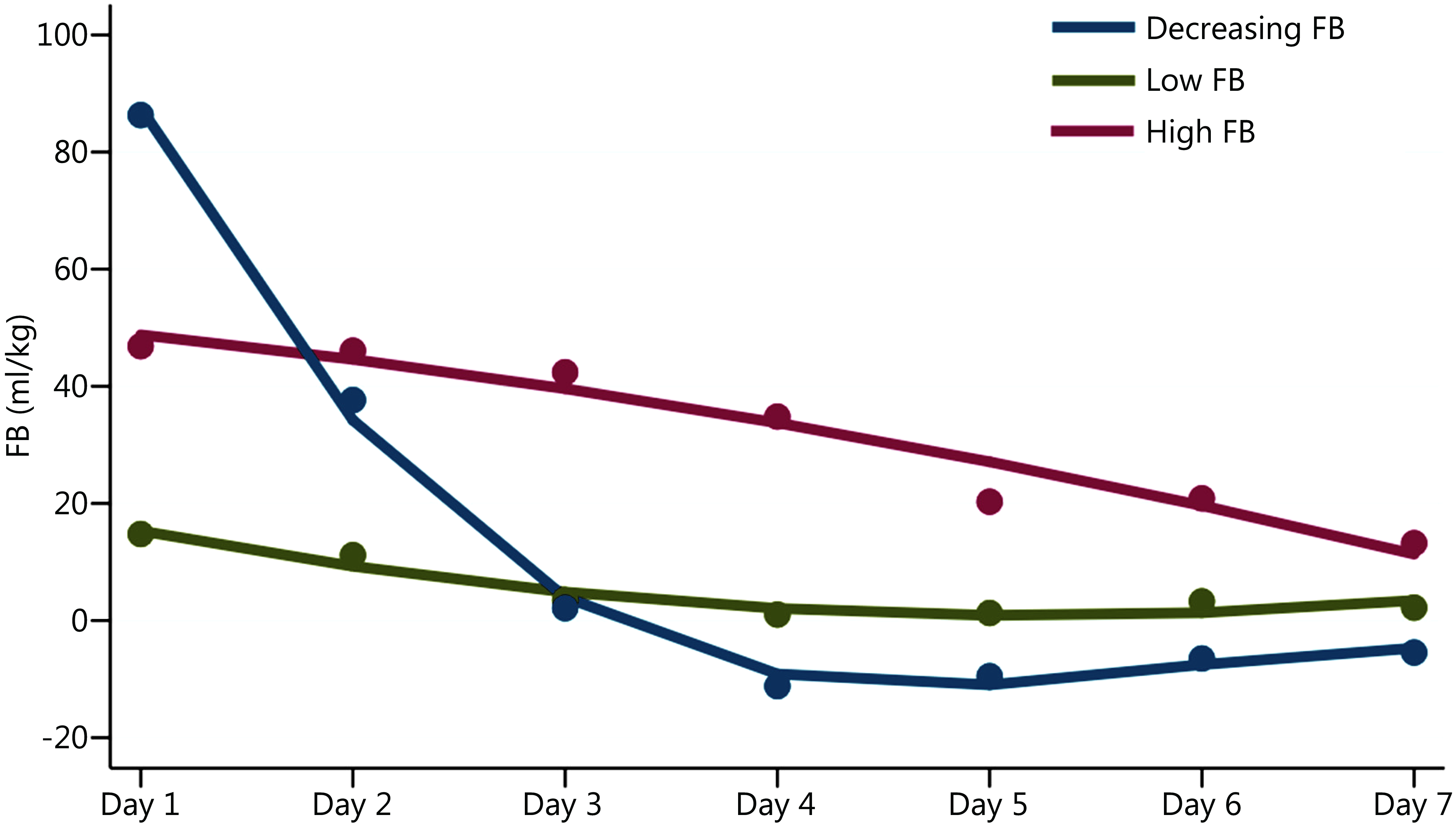

Supplement: Supplementary file 2 — Additional file 2: Fig. S1. Fluid balance trajectory patterns in septic patients without RRT during the first 7 days after ICU admission. [file 40779_2021_328_MOESM2_ESM.tif]
