# Supplementary material for: Association of fluid balance trajectories with clinical outcomes in patients with septic shock: a prospective multicenter cohort study
Source: Mil Med Res. 2021 Jul 6;8:40. doi: 10.1186/s40779-021-00328-1 (PMC8258941; doi:10.1186/s40779-021-00328-1)
Supplement: Supplementary file 3 — Additional file 3: Supplemental file 2. All other ethical bodies that approved our study in the various centers involved. [file 40779_2021_328_MOESM3_ESM.docx]

**All other ethical bodies that approved our study in the various centers involved:**

The institutional review board of Peking Union Medical College Hospital;

The institutional review board of Beijing Tongren Hospital, Capital Medical University;

The institutional review board of Beijing Tiantan Hospital Affiliated to Capital Medical University;

The institutional review board of Beijing Friendship Hospital, Capital Medical University;

The institutional review board of Xuanwu Hospital, Capital Medical University;

The institutional review board of Beijing Chaoyang Hospital, Capital Medical University;

The institutional review board of Peking University Third Hospital;

The institutional review board of China-Japan Friendship Hospital;

The institutional review board of the First Affiliated Hospital of China Medical University;

The institutional review board of Guangdong General Hospital;

The institutional review board of Xiangya Hospital, Central South University;

The institutional review board of West China Hospital, Sichuan University;

The institutional review board of General Hospital of Ningxia Medical University;

The institutional review board of the First Hospital of Jilin University;

The institutional review board of Zhongshan Hospital, Fudan University.
